# Pragmatic Return to Effective Dental Infection Control through Triage and Testing (PREDICT): an observational, feasibility study to improve dental office safety

**DOI:** 10.1186/s40814-024-01471-x

**Published:** 2024-02-28

**Authors:** Janine Fredericks-Younger, Cecile A. Feldman, Veerasathpurush Allareddy, Ellen Funkhouser, MaryAnn McBurnie, Cyril Meyerowitz, Pat Ragusa, Julie Chapman-Greene, Modupe Coker, Daniel Fine, Maria Laura Gennaro, Gayathri Subramanian

**Affiliations:** 1 School of Dental Medicine, Rutgers University, Office of Academic Affairs, 110 Bergen Street, Rm B813, Newark, NJ 07103 USA; 2 School of Public Health, Rutgers University, Office of Academic Affairs, 110 Bergen Street, Rm B813, Newark, NJ 07103 USA; 3 College of Dentistry, University of Illinois Chicago, Urbana, IL USA; 4 University of Alabama, Tuscaloosa, AL USA; 5 Kaiser Permanente, Center for Health Research, Portland, OR USA; 6 Eastman Institute for Oral Health, University of Rochester, Rochester, USA; 7 New Jersey Medical School, PHRI Center, Rutgers University, Office of Academic Affairs, 110 Bergen Street, Rm B813, Newark, NJ 07103 USA

**Keywords:** SARS-CoV-2, Infection control, Dental, COVID-19 testing, Feasibility studies, Dental offices, Health personnel, Dental care, Continuity of patient care, Workforce, Perception

## Abstract

**Background:**

During the COVID-19 pandemic, there was a substantial interruption of care, with patients and workers fearful to return to the dental office. As dental practice creates a highly aerosolized environment, the potential for spread of airborne illness is magnified. As a means to increase safety and mitigate risk, pre-visit testing for SARS-CoV-2 has the potential to minimize disease transmission in dental offices. The Pragmatic Return to Effective Dental Infection Control through Testing (PREDICT) Feasibility Study examined the logistics and impact of two different testing mechanisms (laboratory-based PCR viral testing and point-of-care antigen testing) in dental offices.

**Methods:**

Dental healthcare workers (DHCWs) and patients in four dental offices within the National Dental Practice-based Research Network participated in this prospective study. In addition to electronic surveys, participants in two offices completed POC testing, while participants in two offices used lab-based PCR methods to detect SARS-CoV-2 infection. Analysis was limited to descriptive measures, with median and interquartile ranges reported for Likert scale responses and mean and standard deviation for continuous variables.

**Results:**

Of the total 72 enrolled, 28 DHCWs and 41 patients completed the protocol. Two patients (4.9%) tested positive prior to their visit, while 2 DHCWs (12.5%) tested positive for SARS-CoV-2 infection at the start of the study. DHCWs and patients shared similar degree of concern (69% and 63%, respectively) for contracting COVID-19 from patients, while patients feared contracting COVID-19 from DHCWs less (49%). Descriptive statistics calculations revealed that saliva, tongue epithelial cells, and nasal swabs were the most desirable specimen collection method; both testing (LAB and POC) protocols took similar amounts of total time to complete; and DHCWs and patients reported feeling more comfortable when both groups were tested.

**Conclusions:**

While a larger-scale, network study is necessary for generalizability of results, this feasibility study suggests that SARS-CoV-2 testing can be effectively implemented into dental practice workflows and positively impact perception of safety for DHCWs and patients. As new virulent infectious diseases emerge, preparing dental personnel to employ an entire toolbox of risk mitigation strategies, including testing, may have the potential to decrease dental practice closure time, maintaining continuity of dental care services for patients.

**Trial registration:**

ClinicalTrials.gov: NCT05123742.

## Key messages regarding feasibility

### What uncertainties existed regarding feasibility?

It is unclear why dentistry did not embrace routine pre-visit SARS-COV-2 testing like other medical professions at the start of the COVID-19 pandemic. We sought to understand:

- Would DHCWs and patients be willing to undergo infectious disease testing as an effort to mitigate risk?
- Would testing impact perception of safety within the dental office during this time?
- Could infectious disease testing be effectively implemented within dental practice workflows considering resources required and who would bear the cost?

### What are the key feasibility findings?

- Dental offices could effectively implement infectious disease testing in the dental practice workflows for both staff and patients.
- Both DHCWs and patients were willing to be tested and testing positively impacted perception of safety.
- Patients would be willing to pay additional fees (approximately $15) for testing

### What are the implications of the feasibility findings for the design of the main study?

- In designing a larger-scale, network study, we aim to identify best practices for implementing infectious disease testing as a high impact risk mitigation strategy, which can be employed in future pandemics.
- Testing could maximize the safety of DHCWs and patients from novel infections, while reducing office closures and workforce exodus.

## Background

The emergence of the severe acute respiratory syndrome coronavirus 2 (SARS-CoV-2 virus) has reminded the world of the dangers humanity faces from novel infections [1, 2]. First identified in December 2019 [3], the SARS-CoV-2 virus upended our day-to-day activities, initially driving many into isolation, causing interruptions in the provision of essential medical and dental care [4–10]. As the disease shifts from pandemic to endemic [11], SARS-CoV-2 continues to mutate [12, 13]. While initially virulent with high mortality, the virus is less virulent though morbidity and mortality remains high [14, 15].

Throughout history, the emergence of novel pathogens have changed practice patterns and will continue to challenge and alter current healthcare practice. For example, in the late 1980s, the human immunodeficiency virus significantly impacted personal protective equipment (PPE) standards [16, 17]. More recently, the halting of routine dental care services at the start of the coronavirus disease of 2019 (COVID-19) pandemic illustrated the need to have mechanisms in place to mitigate risk and ensure continued safety of dental healthcare workers (DHCWs) and patients [18–20]. Infectious disease testing is one important measure that can potentially curtail interruptions in care. Several months after the start of the pandemic, polymerase chain reaction (PCR) and antigen point-of-care (POC) testing became available [21]. Hospitals and medical offices quickly incorporated these testing technologies into their practice workflows to increase safety and minimize in-office disease transmission for staff and patients [22–24]. Dental practices, however, were slow to adopt this mitigation strategy [25], despite the nature of dental procedures, many being aerosol generating using of high-speed handpieces and ultrasonic scalers, compounding the potential risk of SARS-CoV-2 transmission within the dental office [26–32].

When a virus with high morbidity and/or mortality is widely circulating within a community, testing is a key mitigation strategy that should be considered within dental practice to help prevent in-office transmission [33–35]. By identifying infected individuals and halting person-to-person contact through testing, both DCHWs and patients can have an increased perception of safety within the dental practice environment [33, 34].

The Pragmatic Return to Effective Dental Infection Control through Triage and Testing (PREDICT) Feasibility study [36] was designed to examine the feasibility for implementing two different COVID-19 testing strategies [lab-based polymerase chain reaction (PCR) and point-of-care testing (POC)] in dental offices. PREDCIT sought to identify advantages and potential barriers for each testing method and evaluate the impact of each strategy on patients’ and DHCWs’ perceptions of safety in dental offices. Study aims included the following: (1) to determine DHCW and patient willingness to participate; (2) to determine DHCW and patient willingness/ability to follow thru with triage, testing, and survey administration procedures; and (3) to determine ease of use of electronic survey instruments for both the DHCWs and patient participants. Results of this feasibility study provided preliminary data to inform the development of a large, network-wide study that seeks to identify key mitigation strategies to can prevent SARS-CoV-2 or other novel infectious agents that may affect safety and perception of safety in a dental office setting, to ultimately maintain a willing workforce and minimize interruptions to essential dental care for patients.

## Methods

The PREDICT Feasibility Study was an observational study conducted within the National Dental Practice-Based Research Network (PBRN). Funded by the National Institute of Dental and Craniofacial Research of the National Institutes of Health, the PBRN consists of over 7000 dental professionals across the United States who collaborate and conduct practice-based research [37, 38]. Within this rich and diverse network, members are committed to advancing knowledge of dental practice by pursuing pragmatic approaches to answer important clinical questions [39, 40]. Practice-based research explores answers to questions in actual practice environments where patient and dental provider preferences and biases influence decisions and outcomes.

### Participants

The PREDICT Feasibility Study required participation of DHCWs and patients within dental practices. Four clinician investigators in the National Dental PBRN were recruited to participate. Each of the investigators worked within a dental office/practice with at least five DHCWs, each of which had the option to engage in or decline participation. All interested DHCWs in each of the four offices were consented by a PBRN Research Coordinator. All DHCW and patient participants were selected based on the following inclusion criteria: over 18 years of age, able to understand English, and able to sign consent. Exclusion criteria included individuals who previously participated in a prior COVID-19 testing feasibility study.

### Interventions

**Fig. 1:**
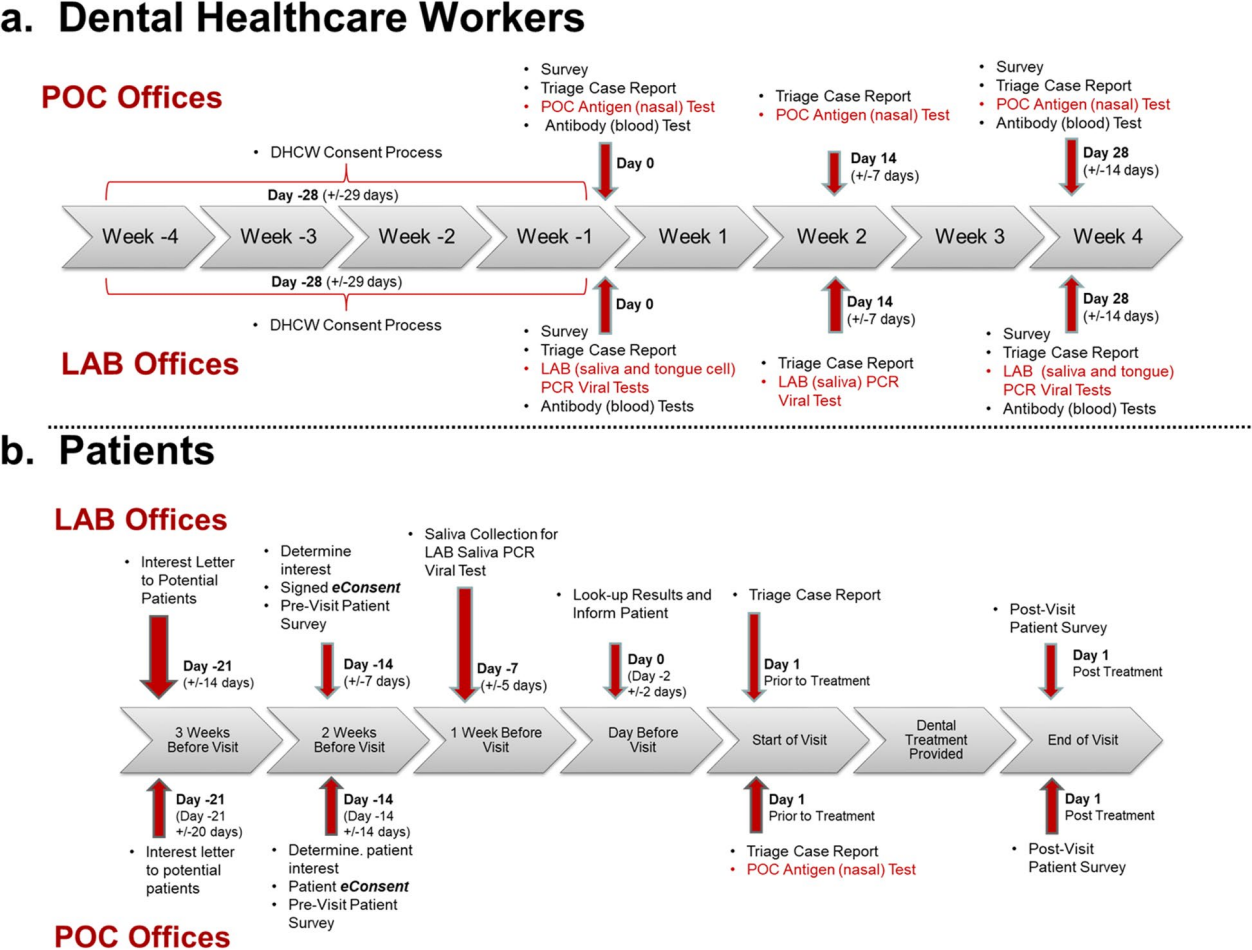
PREDICT Protocols: LAB and POC protocols for dental healthcare workers and the LAB and POC protocols for patient participants

There were two testing groups for DHCWs and patients: POC and LAB. Two offices were designated as LAB offices implementing the LAB protocol (PCR testing) for DHCWs and patients, while two offices were designated as POC offices testing the POC protocol. In total, four protocols were developed. Figure 1a illustrates the LAB and POC protocols for DHCWs, and Fig. 1b illustrates the LAB and POC protocols for patients.

- DHCW–LAB protocol: DHCWs were consented by the PBRN Research Coordinator. On day 1 (start-of-study), DHCW participants completed a start-of-study survey, a symptom triage report and collected saliva, tongue epithelium, and capillary blood samples which were sent to the lab for processing. Two weeks later, a second symptom triage report was completed, and saliva and tongue specimens were collected. Finally, 2 weeks later, a third symptom triage report was completed; saliva, tongue epithelium cells, and capillary blood sample collected along with completion of an end-of-study survey and study feasibly survey. SARS-CoV-2 test results along with antibody IgG and IgM results were made available to the PBRN investigator for sharing with dental office personnel as soon as available from the viral and antibody processing laboratories.
- DHCW–POC protocol: DHCWs are consented by the PBRN Research Coordinator. On day 1 (start-of-study), DHCW participants completed a start-of-study survey, a symptom triage report, performed the POC SARS-CoV-2 antigen test, and provided capillary blood samples. Two weeks later, a second symptom triage report was completed and with the POC test repeated. Finally, 2 weeks later, a third symptom triage report was completed; POC test and capillary blood specimen collection repeated along with completion of an end-of-study survey and study feasibly survey.
- PATIENT-LAB protocol: After written informed consent was obtained by a PBRN investigator, patients were asked to complete pre-visit questionnaire and were sent salvia collection kits. One week prior to their visit patients were requested to collect their saliva sample and drop the sample off at their dental office, which then forwarded the sample to the lab for analysis. Lab results were forwarded to the PBRN practitioner to inform patient participants prior to their dental visit. The symptom triage report was completed upon reporting for their dental visit. At the completion of their dental visit, a post-visit survey was completed along with a study feasibly questionnaire.
- PATIENT-POC protocol: After written informed consent was performed by a PBRN investigator, patients were asked to complete a pre-visit questionnaire. Upon reporting for their visit, a symptom triage report was completed and the POC SARS-CoV-2 antigen test completed. At the completion of their dental visit, a post-visit survey was completed along with a study feasibly questionnaire.

The Abbott BinaxNOW test SARS-CoV-2 was utilized within POC offices. A nasal swab specimen was collected and inserted into the Abbott BinaxNOW COVID-19 antigen card to test for nucleocapsid protein antigen, which is used to determine SAR-CoV-2 infection. Conversely, in LAB offices, saliva and tongue samples were obtained and sent to the University lab for processing. Specifically, genetic material was extracted from the saliva and tongue epithelium samples via polymerase chain reaction (PCR) test to detect the presence of SARS-CoV-2 RNA, which can indicate present or past SARS-CoV-2 infection.

In addition to testing, questionnaires and surveys were administered to all participants electronically using Research Electronic Data Capture (REDCap), a secure web application, which supports research data collection and operations. For DHCWs, questionnaires at the start and end of the study assessed the impact of regular testing on the perception of safety at two-week intervals. Start of study questions included demographics, PPE used in the office, work practice controls used in the office, importance of triage and testing, importance of PPE measures, perceptions of safety and comfort in the workplace, safety culture in the office, SARS-CoV-2 testing preferences, dentist’s role in SARS-CoV-2 testing, and willingness to test in the office. The DHCW End of Study Survey included questions related to the importance of triage and testing, importance of PPE measures, perceptions of safety and comfort in the workplace, safety culture in the office, SARSCoV-2 testing preferences, dentist’s role in SARS-CoV-2 testing, willingness to test in the office, and vaccinations. The DHCW Participation Survey explored perceptions related to study participation including survey and testing logistics.

Similarly, patient pre- and post-visit patient questionnaires examined their beliefs and attitudes pre- and post-visit. The Patient Pre-visit Questionnaire investigated perceptions of safety and comfort, reasons for delaying dental care, concerns about returning to dental care, safety precautions valued, importance of triage and testing, and demographics. The Patient End-of-Visit Survey explored perceptions with testing preferences, PPE observed, environmental controls observed, concerns about returning to dental care, safety precautions valued, importance of triage and testing, likelihood of reporting symptoms, dentist’s role in COVID-19 testing, and vaccinations. The Patient Participation Survey probed perceptions related to study participation including ease of survey and testing logistics.

### Outcome measures and statistical analysis

Outcome measures included both process and “effect of intervention” impact measures. Process measures included time required to collect and process specimens, ability to complete the protocol within each window, length of time between obtaining the specimen and obtaining the results, and ability to obtain SARS-CoV-2 viral and antigen results prior to start of the dental visit. Effect of intervention outcomes included sense of safety using the numeric rating scale (NRS), if (1) patients are tested, (2) DHCWs are tested, and (3) both patients and DHCWs tested. Data related to specimen and test type preferences, willingness and amount to pay, and required specificity and sensitivity levels were also collected as responses could significantly impact the design of testing protocols in dental offices.

For this feasibility study, analysis was limited to descriptive measures. Median and interquartile ranges were reported for Likert scale responses and mean and standard deviation for continuous variables. Separate results were reported for DHCWs and patients as protocols differed slightly for the groups (e.g., patients were queried before and after their visit and DHCWS were queried 3 times at 2-week intervals). Comparisons between LAB and POC DHCWs and patients were not performed as the number of participants was limited. Descriptive statistics were calculated using JMP Pro 16 and SAS.

### Ethical considerations

Human subject protection review was conducted and approved by the University Institutional Review Board. As PBRN investigators were dentists, testing was performed as screening for SARS-CoV-2, not to definitively diagnose COVID-19. Investigators were encouraged to refer positive patients or DHCWs to their primary care providers.

## Results

### Study participants

**Fig. 2:**
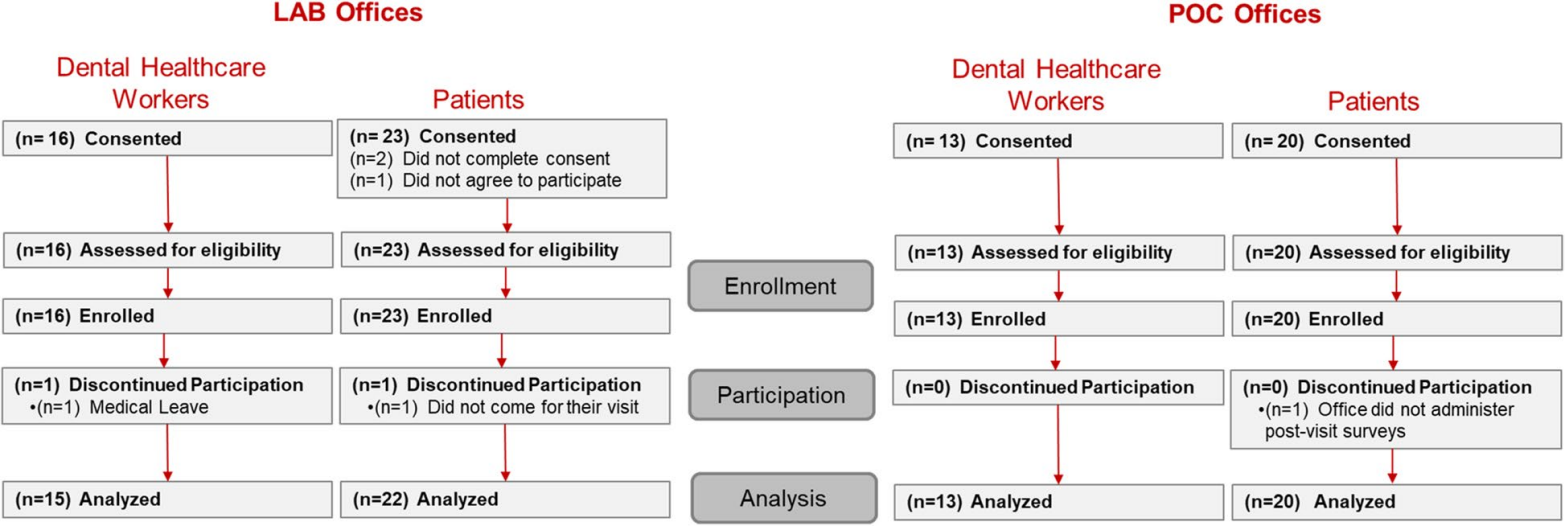
Participant enrollment and completion status for dental healthcare worker and patient participants in the LAB and POC designated offices

Over a 3-month period (December 2021–February 2022), the four participating offices completed the recruitment objectives of a minimum of 5 DHCWs and 10 patients. In total, 29 DHCWs and 43 patients were consented (Fig. 2), with 28 DCHWs and 41 patients completing the protocol. One DHCW was lost due to a non-COVID-19-related illness. One patient was consented but failed to report for their clinical visit, while another patient did not complete the post-visit surveys.

Participant demographics (Table 1) from the four pilot offices revealed that majority of the DHCW participants were Caucasian (86%) and non-Hispanic (79%). Similarly, patient participants were predominantly Caucasian (88%) and non-Hispanic (84%). The majority of participants lived in suburban surroundings with most DHCWs and patients having a household income of more than $100,000. Mean age of DHCWs was 50, while the mean age of patients was 57. The twenty-nine DHCW participants had varied practice roles with the majority working chairside including twelve (41%) dental assistants, five (17%) owner dentists, four (14%) associate dentists, and five (17%) dental hygienists. The majority of DHCW (90%) had some college education with twelve (41%) having participated in graduate level education. The majority of patient participants had engaged in college level coursework (30% completing some college, 35% Bachelor’s degree, and 21% with a graduate degree).

**Table 1:** Participant demographics

	**DHCWs**	**Patients**
	***n* = 29** **Median (IQR)**	***n* = 43** **Median (IQR)**
**Age**		
Years	50 (32–62)	57 (38–67)
**Gender**	***N* (%)**	***N* (%)**
Male	5 (17%)	16 (37%)
Female	24 (83%)	27 (63%)
**Race**	***N* (%)**	***N* (%)**
American Indian or Alaskan Native	0 (0%)	0 (0%)
Asian	0 (0%)	1 (2%)
Native Hawaiian or Other Pacific Islander	0 (0%)	0 (0%)
Black or African American	0 (0%)	2 (5%)
White or Caucasian	25 (86%)	38 (88%)
More than one race	1 (3%)	0 (0%)
Not provided (prefer not to answer)	3 (10%)	2 (5%)
**Ethnicity**	***N* (%)**	***N* (%)**
Hispanic	3 (10%)	6 (14%)
Non-Hispanic	23 (79%)	36 (84%)
Not provided	3 (10%)	1 (2%)
**Household Income**	***N* (%)**	***N* (%)**
< 25 K	0 (0%)	1 (2%)
25–50 K	0 (0%)	2 (5%)
50–100 K	8 (28%)	10 (23%)
> 100 K	14 (48%)	20 (46%)
Not provided (prefer not to answer)	7 (24%)	10 (23%)
**Education**	***N* (%)**	***N* (%)**
Less than high school diploma	0 (0%)	0 (0%)
High school/GED	3 (10%)	5 (12%)
Some college	13 (45%)	13 (30%)
Bachelor	1 (3%)	15 (35%)
Graduate	12 (41%)	9 (21%)
Not provided	0 (0%)	1 (2%)
**Community**	***N* (%)**	***N* (%)**
Urban	5 (17%)	4 (9%)
Suburban	14 (48%)	26 (60%)
Rural	10 (34%)	11 (26%)
Not provided	0 (0%)	2 (5%)
**Job within Dental Office**	***N* (%)**	***N* (%)**
Dentist, owner	5 (17%)	n/a
Dentist, associate	4 (14%)	n/a
Hygienist	5 (17%)	n/a
Dental assistant	12 (41%)	n/a
Receptionist/financial/other	3 (10%)	n/a

### COVID-19 history and concern of contracting COVID-19 (Table 2)

**Table 2:** COVID-19 history and concern of contracting COVID-19 for DHCW and patient participants

	**DHCWs** ***N*** ** = 29**	**Patients** ***N*** ** = 43**
**COVID-19 history**	***N*** ** (%) indicating yes**	***N*** ** (%) indicating yes**
Ever been diagnosed with COVID	7 (24%)	12 (28%)
Anyone you live with diagnosed with COVID	4 (14%)	14 (33%)
Anyone you work with diagnosed with COVID	21 (72%)	n/a
Vaccinated against COVID	25 (86%)	40 (93%)
**Concern contracting COVID-19 from patients**	***N*** ** (%)**	***N*** ** (%)**
Not at all	9 (31%)	16 (37%)
Mild	11 (38%)	16 (37%)
Moderate	7 (24%)	9 (21%)
Severe	2 (7%)	2 (5%)
**Concern contracting COVID-19 from staff**	***N*** ** (%)**	***N*** ** (%)**
Not at all	9 (31%)	22 (51%)
Mild	14 (48%)	10 (23%)
Moderate	5 (17%)	9 (21%)
Severe	1 (3%)	2 (5%)

One-fourth of DHCWs and patients reported a history of a positive COVID-19 diagnosis prior to participation (Table 2). Patients reported more household members having been diagnosed with COVID-19 than DHCWs. Almost three quarters of DHCWs reported office mates having been diagnosed with COVID-19. Related to vaccinations, 86% of DHCWS and 93% of patients reported having received at least the first dose of a COVID-19 vaccination.

Related to perceptions of transmissibility within the dental office environment, 69% of DHCWs and 63% of patients indicated some degree of concern of contracting COVID-19 from patients in the dental office environment. Similarly, 69% of DHCWs and 49% of patients had some degree of concern of contracting COVID-19 from dental office personnel, with patients concerned to a lesser extent.

### COVID-19 test results

Of the 41 patient participants, two tested positive for SARS-CoV-2 infection: one patient in the POC group (5.0%) and one in the LAB group (4.8%). For the 28 DHCWs who tested three times within a 4-week period, several DHCWs tested positive for SARS-CoV-2 infection. At both the start and end of the study, there was at least one DHCW identified as positive to SARS-CoV-2 through PCR (LAB). At the start of the study, two workers were identified as SARS-CoV-2 positive (12.5%) through PCR testing using both saliva and tongue specimens, though both workers were asymptomatic and reported having been diagnosed with COVID-19 several weeks prior. At the end of study, PCR testing results varied by specimen type as PCR testing using tongue epithelial cells identified three cases, whereas PCR testing using saliva specimens did not for the same individual.

### COVID-19 testing preferences (Table 3)

**Table 3:** SARS-CoV-2 testing preferences for DHCW and patient participants

**Testing preference outcomes**				
	**DHCWs** ***N*** **= 28**		**Patients** ***N*** **= 41**	
**Specimen preference (1 (most) to 6 (least preferred))**	**Median (IQR)**		**Median (IQR)**	
Saliva	1 (1–2)		2 (1–3)	
Tongue epithelial cells	2 (1–3)		2 (1–3)	
Nasal swab	2.5 (2–3)		2 (1–3)	
Nasal pharyngeal swab	4 (4–4)		4 (3–5)	
Finger stick	5 (5–5)		5 (4–5)	
Venous	6 (6–6)		6 (6–6)	
**Testing protocol preference (1 (most) to 4 (least preferred))**	**Median (IQR)**		**Median (IQR)**	
Home test and mail to lab	2 (1–2.5)		1 (1–2)	
POC in office	1.5 (1–3)		2 (1–3)	
Specimen to dental office and then to Lab	2 (2–3)		3 (2–3)	
Specimen to commercial lab	4 (3.5–4)		4 (4–4)	
**How safe do you think you would feel if…**	**Median (IQR)**		**Median (IQR)**	
All DHCW regularly test AND all patients tested prior to dental visit	85.5 (67–100)		97 (88–100)	
Just DHWS are regularly tested	73.5 (50–88.5)		79 (65–97)	
Just patients are regularly tested	54.5 (50–85)		55 (37–78)	
**Cost willing to pay for COVID test**	**Median (IQR)**		**Median (IQR)**	
DHCW testing	$10 ($0–$20)		n/a	
Patient testing	$18 ($10–$36)		$15 ($9–$25)	
**Desired test specificity and sensitivity (scale 0 to 100)**	**Median (IQR)**			
Lowest acceptable sensitivity (limited to DMD/DDSs) *N* = 9	90 (89–98)		n/a	
Lowest acceptable specificity (limited to DMD/DDSs) *N* = 9	90 (89–95)		n/a	
**If you had a choice which office would you prefer to go to:**			***N*** ** (%)**	
Where patients and staff are tested	n/a		31 (76%)	
Does not make a difference if patients and staff are tested	n/a		10 (24%)	
Patients and staff are NOT tested	n/a		0 (0%)	
**Testing process outcomes**				
	**PCR**	**POC**	**PCR**	**POC**
**Ease of performance**	***n*** **= 15** ***N*** **(%) Indicating Easy**	***n*** **= 12** ***N*** **(%) Indicating Easy**	***n*** **= 22** ***N*** **(%) Indicating Easy**	***n*** **= 19** ***N*** **(%) Indicating Easy**
Collection of nasal specimen	n/a	10 (83%)	n/a	19 (100%)
Processing of nasal specimen	n/a	7 (58%)	n/a	n/a
Collection of saliva specimen	14 (93%)	n/a	21 (95%)	n/a
Preparing and packaging for shipment saliva specimen	14 (93%)	n/a	n/a	n/a
Collection of tongue specimen	14 (93%)	n/a	n/a	n/a
Preparing and packing for shipping tongue specimen	13 (87%)	n/a	n/a	n/a

Both DHCWs and patients found venous blood was the least desirable specimen collection method for COVID-19 testing. Saliva, tongue epithelial cells, and nasal swabs were rated the most desirable specimens for testing. DHCWs preferred POC testing in the dental office. Conversely, patients preferred collecting their specimen at home and mailing the specimen to a lab for processing with POC testing in the dental office were slightly less desirable. Dropping off collected specimens at the dental office and going to a commercial lab for specimen collection and laboratory processing was the least desirable method.

Overall, both participants reported feeling more comfortable being in an office with both DHCWs and patients being tested. Feeling of safety ratings decreased for both groups of participants when testing was limited to DHCWS and decreased even more significantly when testing was limited to patients only. When patients were asked if they preferred to go to an office where COVID-19 testing was regularly performed, 75% reported a preference to going to an office where patients and staff are tested. DHCWs were willing to require patients to pay a median rate of $18 for testing, while patients were willing to pay median rate of $15 to be tested. DHCWs were only willing to pay a median rate of $10 for their own testing.

When asked about minimum levels of sensitivity and specificity, DHCWs reported that tests needed to have a specificity and sensitivity of 85% or higher along with at false positives and false negatives being no greater than 50%.

### Screening process outcomes

All screening processes, including specimen collection, and preparation and preparing specimens for shipping, and POC processing were considered easy to perform. Saliva specimen collection (5–10 min) took longer than nasal POC specimen collection (5 min). Both the LAB and POC protocols took similar amounts of total time (approximately 15 min) once all aspects of testing were included (specimen collection, drop off time for PCR testing, and processing time for POC testing).

## Discussion

This feasibility study suggests the following: (1) dental offices can effectively implement SARS-CoV-2 testing into their practice workflows, (2) DHCWs and patients are willing to participate in a SARS-CoV-2 testing program, and (3) this testing mitigation strategy can positively influence the perception of safety within an office environment. Results from this study demonstrate that either testing method, lab-based PCR or POC COVID-19 testing, can in fact be effectively built into dental practice workflows. Sixty-nine of the 72 enrollees completed the protocol including all testing requirements, with three non-completes unrelated to the burden of testing. Both DHCWs and patients reported feeling safer when both dental office personnel and patients were regularly tested. In addition, the majority of patient respondents prefer to go to an office where patients and staff are regularly tested. The identification of individuals positive for SARS-COV-2 infection during this study further demonstrates that the implementation of a SARS-CoV-2 testing program can serve as effective mitigation strategy to decrease in-office transmission.

There are several limitations to this study. As the purpose of this study was to develop the methodology for a large-scale PBRN-based study, the sample size is small, and results are not generalizable. There was limited diversity demographically, geographically, and economically within the population tested. In addition, this feasibility study was conducted in an area of the US that is highly vaccinated and potentially more accepting of testing. Conclusions related to testing preference results are also limited as participants may not have been familiar nor had experience with all testing types. Another limitation specifically related to the logistics of PCR testing. While patients reported a preference for this protocol (specimen collection at-home and mail to lab), the management of this method for the dental office proved difficult. Continuity of care was hampered as results were not readily available. Lab processing efficiency impacted the receipt of timely results for patients with pending dental appointments. At the height of the pandemic, study laboratories engaged in PCR analysis had difficulty keeping up with demand and results were delayed. For dental providers who wish to implement PCR testing, perhaps a more viable method would be to require patients bear full responsibility for scheduling/completing testing at a commercial laboratory and reporting results to the dental office prior to treatment. Another alternative is to select the in-office POC testing method, which eliminates the need for outside processing and analysis.

In choosing a testing method, POC testing has multiple advantages over lab based testing, including ease of use, low cost, and reliability. POC test kits are relatively inexpensive, within the range of willingness to pay, and provide results within 15 min. POC testing also has the added benefit of limiting the number of false positives as PCR testing picks up remnant viral particle presence for several months after a patient’s course of disease is over. Practitioners who avail themselves to pre-visit POC testing should be cognizant, however, of some drawbacks. Limitations include testing administration logistics which require longer patient facing time (sample collection and analysis) and potential added operational costs (instrumentation, physical space, and personnel required to enact workflows). A cost analysis should be run to establish a compensatory cost estimate and the cost–benefit ratio of testing to potential missed appointments or work due to illness must be considered. Overall, in times of high prevalence with high morbidity and mortality, POC testing to mitigate risk of transmission may be an effective strategy to maintain a steady workforce, reduce office closures, and avoid disruption in dental care services.

## Conclusions

DHCWs and patients share concern about transmission of COVID-19 in the dental office and are receptive to SARS-CoV-2 testing as a mitigation strategy. It is feasible to implement SARS-CoV-2 testing in dental practice workflows. While the SARS-CoV-2 virus is now less virulent, SARS-CoV-2 point-of-care (POC) testing can be used as a model to investigate how dental practices can best prepare for the future. Future studies could contribute to the creation of standard testing practices for dental offices that can be adopted during times of high incidence of COVID-19 as well as for the next novel virus.

## Data Availability

The datasets generated and/or analyzed during the current study are maintained and made available by the National Dental PBRN [ https://www.nationaldentalpbrn.org/resource-sharing/]. This feasibility study is registered on Clinicaltrials.gov NCT05123742. The protocol can be accessed in JMIR Res Protoc. 2022 Aug 31;11(8):e38386. doi:10.2196/38386. PMID: 35944181; PMCID: PMC9439378.

## Abbreviations

COVID-19: Coronavirus disease of 2019
DHCW: Dental healthcare worker
NRS: Numeric rating scale
PBRN: Practice-based research network
PCR: Polymerase chain reaction
POC: Point-of-care
PPE: Personal protective equipment
PREDICT: Pragmatic Return to Effective Dental Infection Control through Triage and Testing study
SARS-CoV-2: Severe acute respiratory syndrome coronavirus 2
